# Microbial contribution to the caloric restriction-triggered regulation of the intestinal levels of glutathione transferases, taurine, and bile acid

**DOI:** 10.1080/19490976.2021.1992236

**Published:** 2021-10-25

**Authors:** András Gregor, Marc Pignitter, Slave Trajanoski, Sandra Auernigg-Haselmaier, Veronika Somoza, Jürgen König, Kalina Duszka

**Affiliations:** a Department of Nutritional Sciences, University of Vienna, Vienna, Austria; b Department of Physiological Chemistry, University of Vienna, Vienna, Austria; c Core Facility Computational Bioanalytics, Medical University of Graz, Graz, Austria; d Leibniz-Institut for Food Systems Biology, Technical University of Munich, Munich, Germany

**Keywords:** Caloric restriction, microbiota, bile acids, taurine, glutathione, intestine

## Abstract

Recently we showed that caloric restriction (CR) triggers an increase in the levels of free taurine, taurine-conjugated bile acids (BA), and other taurine conjugates in intestinal mucosa while decreasing glutathione (GSH) levels in wild-type male mice. In the current project, we decided to investigate whether the microbiota is involved in the response to CR by depleting gut bacteria. The antibiotics treatment diminished CR-specific increase in the levels of free taurine and its conjugates as well as upregulated expression and activity of GSH transferases (GST) in the intestinal mucosa. Further, it diminished a CR-related increase in BAs levels in the liver, plasma, and intestinal mucosa. Transplant of microbiota from CR mice to *ad libitum* fed mice triggered CR-like changes in MGST1 expression, levels of taurine and taurine conjugates in the mucosa of the ileum. We show for the first time, that microbiota contributes to the intestinal response to CR-triggered changes in BA, taurine, and GST levels.

## Introduction

Gut microbiota is a complex and dynamic entity characterized by its high variability in composition between different hosts. The inter-individual differences are induced by multiple factors including childbirth delivery, gender, antibiotic treatments, origin, age, and to a great extent by the diet.^1–5^ The type, amount, and timing of the meals are all factors shaping the microbiota.^6–8^ Accordingly, caloric restriction (CR) affects gut bacteria^9–14^ and the composition changes seem to mediate some of the beneficial outcomes of CR, including reduced body weight, decreased blood leptin, and insulin levels.^15^ Yet, the mechanism behind and functionality of CR-specific microbiota are not known. The microbiota plays a major role in numerous aspects of the host’s health and disease by modulating the availability of energy and nutrients, as well as signaling molecules, e.g. hormones, neurotransmitters, ligands, etc.; thereby affecting the host on multiple levels.^16^ Among others, microbiota directly interacts with bile acids (BA) and submits them to various modifications, including deconjugation of BAs and taurine.^17^ Multiple bacterial processes result in the generation of secondary BAs, of which the function and signaling vary from the primary BAs.^18^ Reversely, BAs,^19^ similarly to taurine^20,21^ modulate gut bacteria composition. Taurine reduces the growth of harmful bacteria and stimulates the production of short-chain fatty acids.^20^ Taurine increases the number of selected immune cells, total cells in Payer’s patches,^21^ and attenuates induced colitis in the mouse intestine.^22,23^ Besides anti-inflammatory^24–28^ activity taurine has also, antioxidative,^27,29–32^ and osmoprotective^31,33^ properties.

A complex network of enzymes and transcription factors controls BA synthesis and circulation between the liver and intestine. High levels of BAs inhibit the activity of the key enzyme of the classic synthesis pathway, cholesterol 7 α-hydroxylase (Cyp7a1) via farnesoid X receptor (FXR) or fibroblast growth factor 19 (FGF19).^34,35^ FXR is activated by BAs and, besides establishing a negative feedback loop, it regulates the expression of BA transporters and impacts inflammation.^36^ Moreover, FXR also takes input from gut microbiota thereby integrating the connection between BAs, bacteria, and inflammation. Our recent original study indicated that the liver in CR animals shows increased mRNA expression of cysteine dioxygenase (CDO), an enzyme responsible for the metabolism of cysteine to taurine.^37^ The CR animals also show enhanced expression of Cyp7a1, Ntcp (Na^+^/taurocholate cotransporting polypeptide), which are necessary for BA synthesis and transport. Further, we demonstrated that CR results in increased levels of taurine-conjugated BAs in the intestinal content and mucosa.^14,37^ BAs in the intestine undergo deconjugation and this leads to high levels of free taurine. Simultaneously, CR stimulates the expression and activity of glutathione (GSH) S-transferases (GST) that conjugate taurine leading to an increase in GSH-taurine conjugate levels. The occurrence of GSH-taurine conjugate in conjunction with CR triggers enhanced intestinal uptake of taurine.^37^ Knowing the vast role of gut bacteria in the metabolism of BAs, we hypothesized that microbiota may contribute to the CR-triggered phenomena. Here, we present novel results showing that microbiota is required for the CR-triggered phenotype associated with GSH and taurine conjugation as well as intestinal uptake.

## Results

**Figure 1.**
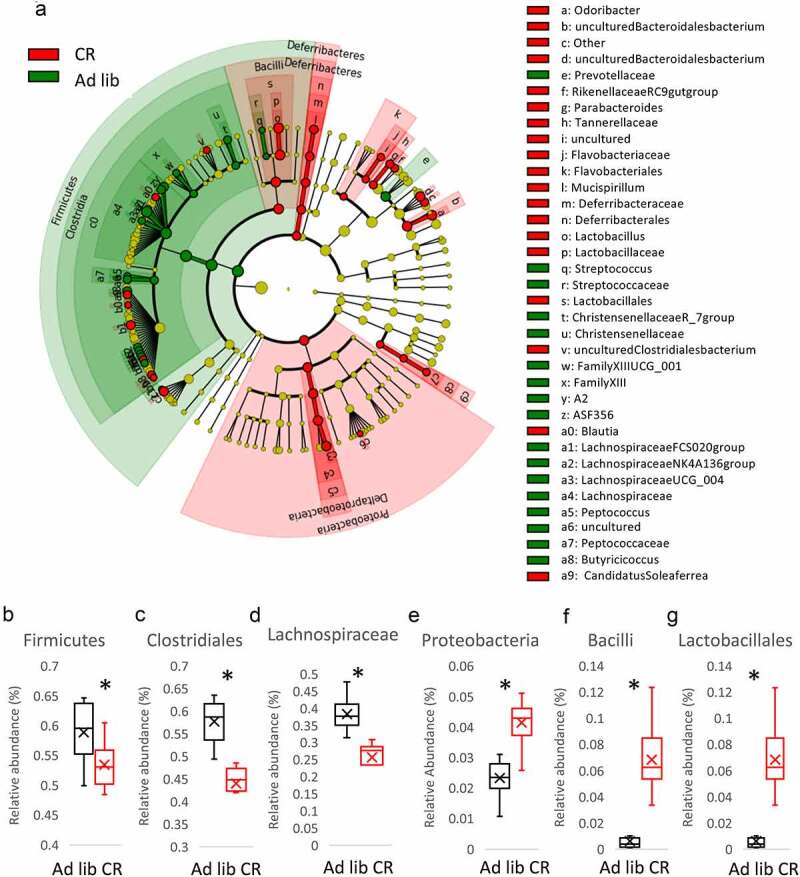
Caloric restriction (CR) modifies microbiota composition in the cecum. Cecum microbiota was sequenced and the results are shown as a Cladogram representing the Linear discriminant analysis Effect Size (LEfSe) results on the hierarchy induced by the taxa. Only significantly changed taxa names with the corresponding color are shown. The figure presents results with a linear determinant analysis (LDA) score bigger than 2 and a *p*-value smaller than .05. (a). The abundance of Firmicutes (b), Clostridiales (c), Lachnospiraceae (d), Proteobacteria (e), Bacilli (f), and Lactobacillales (g) in the cecum of *ad libitum* and CR mice was presented. Two-tailed Student’s t-tests were used to compare the experimental groups of the panels B-G

CR triggers strong microbiota composition changes in the cecum on multiple phylogenic levels (Figure 1a, Suppl fig S1). We researched the impact of taurine on microbiota and compared the published results^20,21^ with our sequencing data of *ad libitum* fed and CR mice cecum. Among the bacteria already known to be affected, we found *Firmicutes, Clostridiales*, and *Lachnospiraceae* downregulated in the CR mice whereas *Proteobacteria, Bacilli*, and *Lactobacillales* were upregulated (Figure 1b-g). Interestingly, the exact opposite regulation was reported after the administration of taurine.^20,21^

**Figure 2.**
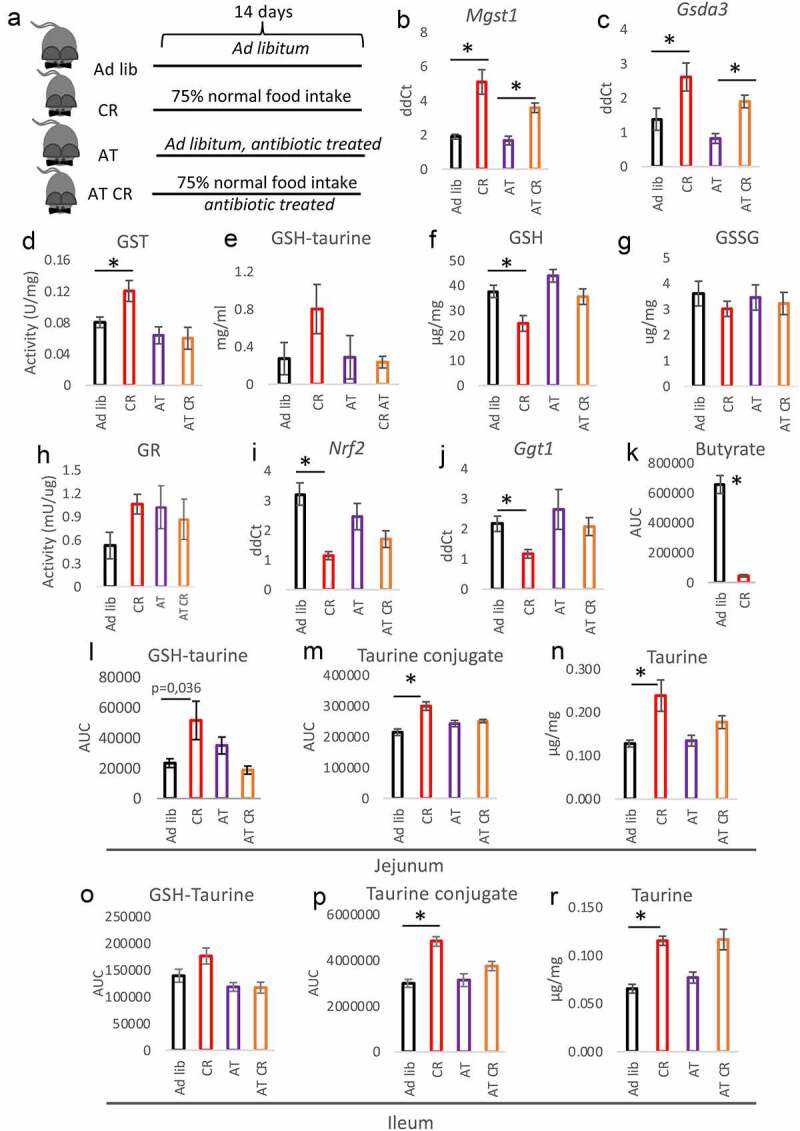
Microbiota depletion neutralizes the CR phenotype in the intestine. The mice were fed *ad libitum* or submitted CR with (AT and AT CR) or without antibiotics treatment (Ad lib and CR) to deplete microbiota (a). Gene expression of the different glutathione-S transferase (GST) subtypes (b-c), and GST activity (d) were measured in the jejunal mucosa. Tissue lysate of jejunum mucosa was incubated with a solution containing GSH and taurine and occurrence of GSH-taurine conjugate was assessed (e). The levels of reduced glutathione (GSH) (f) and oxidized GSH (GSSG) (g) were measured in the jejunal mucosa. The activity of GSH reductase (GR) (h), as well as expression of nuclear factor erythroid 2-related factor 2 (*Nrf2*; I) and γ-glutamyl transpeptidase (*Ggt1*; J) mRNA, was assessed in the mucosa. Butyrate content was quantified in the feces of CR and *ad libitum* mice (k). The levels of GSH-taurine conjugate (l, o), taurine conjugate *m/z* 249 (m, p), and free taurine (n, r) were measured in the jejunum (l-n) and ileum (o-r). Two-tailed Student’s t-tests were used to compare the experimental groups of the panel K. Statistical significance between experimental groups in other panels was evaluated using ANOVA with Bonferroni correction for multiple testing; n = 6–8 (panels B-D and F-R), n = 4 (panel E); *p < .05. Data are presented as the mean±SEM

In order to assess the role of microbiota in the previously described CR-triggered intestinal phenotype,^37^ we applied a mouse model with depleted gut microbiota by treatment with a wide spectrum antibiotic cocktail. We submitted the antibiotics-treated mice to CR (AT CR) and control *ad libitum* feeding (AT) (Figure 2a). In accordance with our earlier report,^37^ CR increases GST (*Mgst1* and *Gsda3*) mRNA expression and enzymatic activity (Figure 2b-c). Antibiotics treatment diminished the CR-induced changes in GST gene expression (Figure 2b-c) and prevented an increase in GST activity (Figure 2d). However, no differences were observed between *ad libitum* and AT *ad libitum* conditions (Figure 2d). CR compared to *ad libitum* conditions increased production of GSH-taurine conjugate when jejunum tissue lysate was incubated with a solution containing taurine and GSH (Figure 2e). Antibiotics treatment neutralized the impact of CR but did not affect the outcomes of *ad libitum* feeding (Figure 2e). Accordingly, the reduction in GSH level in CR compared to *ad libitum* mice was lessened by dosing antibiotics (figure 2f). The concentration of oxidized GSH (GSSG) was not affected by CR or microbiota depletion (Figure 2g). As previously shown,^37^ the CR-triggered imbalance in GSH/GSSG ratio leads to the introduction of glutathione reductase (GR) activity (here not statistically significant after correction for multiple testing) aiming at neutralization of the disparity. The CR animals with depleted intestinal microbiota did not show differences in GR activity compared to *ad libitum* mice (Figure 2h). CR-elicited differences in intestinal mRNA expression of the factors involved in GSH synthesis, NRF2, and GGT1, were offset by bacteria depletion (Figure 2i-j). Searching for the molecular mechanism, we quantified the levels of butyrate, which is one of the short-chain fatty acids produced by microbiota and has been reported to induce GST activity.^38^ However, in CR animals, the amount of cecal butyrate is strongly reduced compared to *ad libitum* mice (Figure 2k).

We reported previously that the levels of oxidative stress-related factors were not affected by CR in the intestinal mucosa.^37^ Similarly, microbiota depletion did not modify it (Suppl fig S2A-B). Nevertheless, the modulation of microbiota neutralized the CR-specific differences in the expression of oxidative stress-related factors catalase, manganese superoxide dismutase (*Mn-Sod*), and thioredoxin 2 (*Trx2*) (Suppl fig S2 C-E).

**Figure 3.**
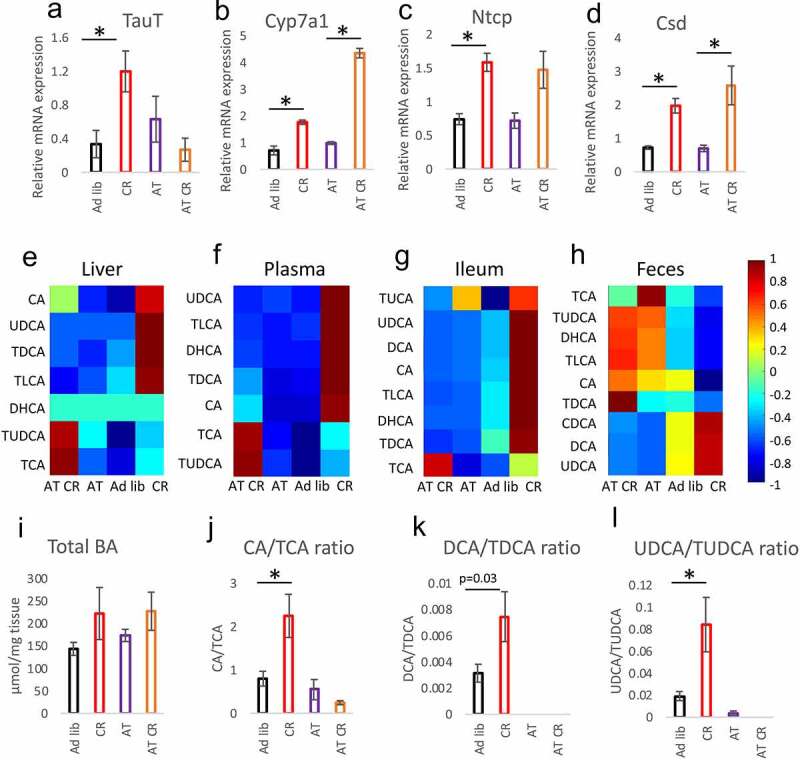
Microbiota and CR jointly regulate the levels of BAs. Gene expression of taurine transporter (TauT) (a), cholesterol 7 α-hydroxylase (Cyp7a1; B), Na^+^/taurocholate cotransporting polypeptide (Ntcp; C), and cysteine sulfonate decarboxylase (Csd; D) was measured in the mucosa of the ileum. The levels of selected bile acids were measured in the liver (e), plasma (f), mucosa of the ileum (g), and feces (h) applying HPLC-MS/MS. Total BAs content was quantified in the mucosa of the ileum using a commercial kit (i). The ratio of CA to TCA (j), DCA to TDCA (k), and UDCA to TUDCA (l) was calculated based on BAs concentration measured in the ileum. Statistical significance between the experimental groups was evaluated using ANOVA with Bonferroni correction for multiple testing; n = 8; *p < .05. Data are presented as mean±SEM. Heatmaps were created by using Z-Scored data, showing the relative deviation from the groups’ mean value, and visualized by using the MATLAB extension COVAIN

As we published before,^37^ CR elevates free taurine and taurine conjugates in the mucosa of jejunum and ileum. In the current study, the levels of GSH-taurine conjugates showed a tendency toward increased levels in CR mice; yet, not statistically significantly. The depletion of microbiota minimized the difference between CR and *ad libitum* fed mice in the mucosa of the jejunum (Figure 2l-n) and ileum (Figure 2o-r, Suppl fig S2 F-I) in terms of GSH-taurine conjugate, other taurine conjugates, and free taurine. Further, the mRNA expression of taurine transporter TauT, which was increased in the ileum of CR mice, was not different between AT and AT CR animals (Figure 3a). In agreement with the expression pattern of TauT and our previous publication,^37^ CR reduced the levels of taurine and its conjugates in the mice’s feces (Suppl fig S2 J-K). In the liver, the expression of genes associated with BA synthesis (*Cyp7a1*) and transport (*Ntcp*) as well as cysteine metabolism (cysteine sulfonate decarboxylase; *Csd*) was regulated by CR and not by microbiota depletion (Figure 3b-d). Importantly, antibiotics treatment did not impact the expression of any of the measured genes in *ad libitum* fed mice.

Concerning BAs, CR consistently increased levels of the measured BAs in the liver, plasma, and ileum but much less in the feces (Figure 3e-h, Suppl table S1) correspondingly to our previous report.^37^ However, the differences in the liver, plasma, and ileum were not statistically significant for each BA. Similarly, despite a visible trend, the differences in the concentrations of total BAs in the ileum of *ad libitum* compared to CR mice lacked statistical significance (Figure 3i). In the ileum, antibiotics lowered TDCA, TLCA, and UDCA levels. In the liver, the treatment decreased TDCA and TLCA but increased TUDCA; whereas in plasma it increased the levels of CA, TCA, and TUDCA (Figure 3e-g, Suppl table S1). Antibiotics blunted the CR-related increase in most BAs in all tissues. However, the levels of CA, TCA, and TDCA remained statistically significantly higher in AT CR compared to AT group in the plasma and of TCA in the ileum (figure 3f-g, Suppl table S1). In general, the AT and AT CR treatments resulted in similar patterns of BAs levels changes in the ileum, liver, and plasma (Figure 3e-g, Suppl table S1). Interestingly, a comparison of the ratios of taurine-conjugated (TCA, TDCA, TUDCA) versus their unconjugated counterparts (CA, DCA, UDCA) in the epithelium of jejunum indicated an increased rate of BAs deconjugation in the CR animals (Figure 3j-l). These differences were not present for AT and AT CR animals due to reduction or depletion of the BAs levels. The differences between *ad libitum* and CR taurine-conjugated and unconjugated BAs ratios were much less pronounced in fecal samples (Suppl fig S3A-C).

**Figure 4.**
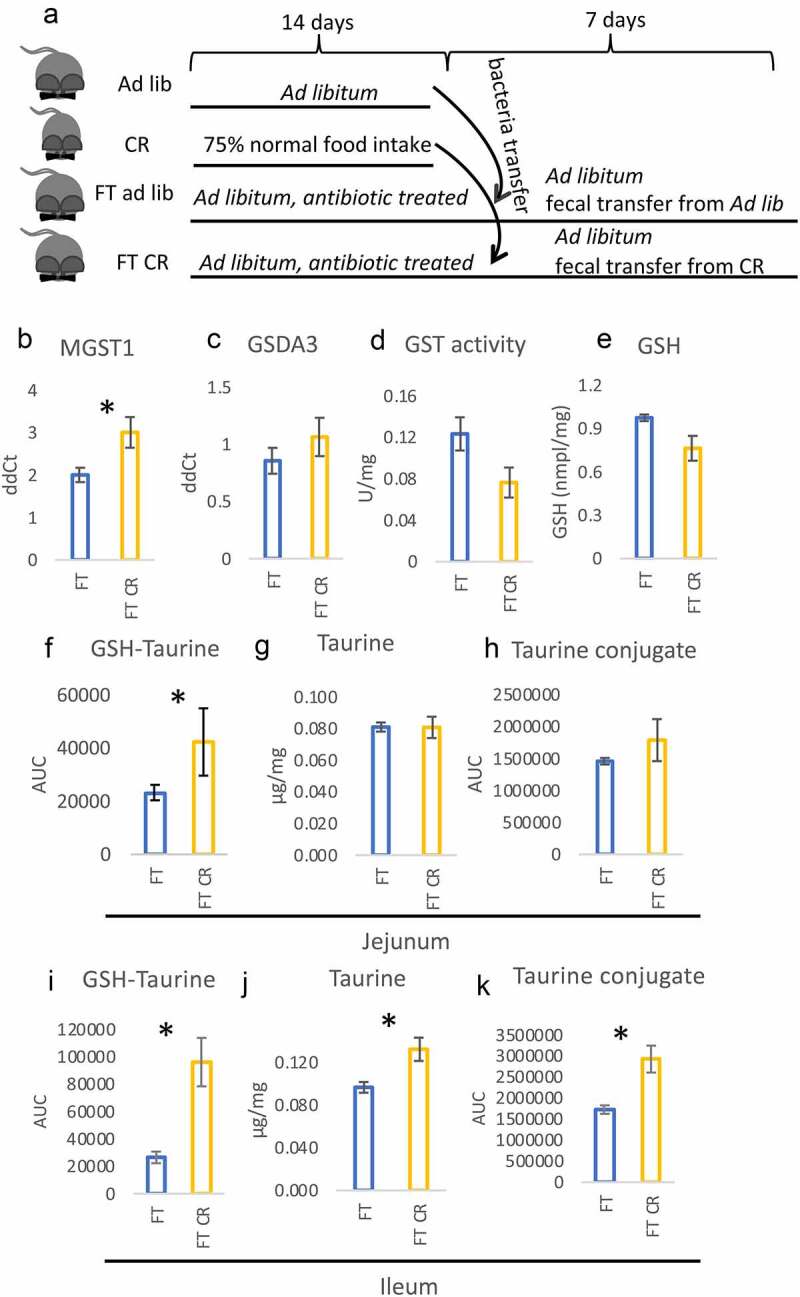
Microbiota can partly induce a CR-like phenotype in the intestine. The mice were fed *ad libitum*, submitted CR, or treated with antibiotics followed by fecal bacteria transfer (FT ad lib and FT CR; A). Gene expression of the different GST subtypes (b-c), GST activity (d), as well as levels of GSH (e), were measured in the jejunal mucosa. The levels of GSH-taurine conjugate (f, i), free taurine (g, j), and taurine conjugate *m/z* 249 (h, k) were measured in the jejunum (f-h) and ileum (i-k). Statistical significance between CR and *ad libitum* groups was evaluated using two-tailed Student’s t-tests; n = 8; *p < .05. Data are presented as mean±SEM

In order to confirm the role of microbiota, we performed fecal transplants from CR and *ad libitum* fed mice to another group of mice with depleted bacteria and fed *ad libitum* (FT, FT CR) (Figure 4a). Microbiota transplant did not change levels of taurine conjugated BAs in the liver, plasma, or epithelium of ileum (Suppl table S1). The CR transplant triggered a CR-like increase in MGST1 gene expression (Figure 4b) but no statistically significant changes in GSDA3 (Figure 4c) expression, GST activity (Figure 4d), or GSH levels (Figure 4e). Notably, FT CR mice experienced a CR-like increase in GSH-taurine conjugates in the jejunum, whereas no changes in the free taurine and taurine conjugates were measured (Figure 4g-h). In the ileum mucosa, the levels of GSH-taurine, free taurine as well as taurine conjugates were increased in FT CR compared to FT mice (Figure 4i-k, Suppl fig S3D-I).

## Discussion

In the previous study, we showed that CR modulates the level of GSH, taurine, and its conjugates as well as the expression and activity of GSTs.^14,37^ Here we demonstrate that the regulation is microbiota-dependent. Crucially, treatment with antibiotics partly neutralizes or completely removes all of the tested parameters affected by CR. The transplant of the microbiota of CR mice to *ad libitum* fed mice reintroduces the CR phenotype to a certain extend. Therefore, the microbiota is a vital part of the response to CR but it requires other CR-related triggers to fully mimic CR. It has previously been reported that microbiota mediates the effect of CR on weight loss, levels of leptin and insulin,^15^ and on metabolic improvements including fat browning, liver health, and better glycemic control.^39^ Additionally, CR suppresses microbial genes for lipopolysaccharides (LPS) synthesis modulating the immune response.^39^ Therefore, the here presented data adds a fitting piece to a picture of microbiota-based response to CR.

The microbiota plays a vital role in the metabolism of BAs and bacterial contribution to the observed phenotype is likely connected with the regulation of the conversion of taurine-conjugated BAs.^40^ We propose a model in which upon increased release of BAs to the intestine during CR, microbiota acts to deconjugate BAs from taurine and therefore raises the levels of free taurine and taurine-deconjugated BAs species. The sudden increased appearance of bioactive taurine requires mechanisms to regulate its availability and activity, which may be executed by GSH, leading to the generation of taurine-GSH conjugates. Interestingly, microbiota transplant triggered greater changes in the ileum compared to the jejunum which may be explained by increasing bacterial abundance along the small intestine and greater capacity of the intestine to take up taurine due to high expression of the taurine transporter TauT in the distal intestine.^41^ Notably, maintenance of proper taurine levels contributes to increased BA formation and secretion and thereby to the regulation of cholesterol levels.^42^ Furthermore, the enhanced BAs and taurine uptake in the CR intestine previously reported by us^14^ likely contributes to diminished or reversed differences for BAs and taurine respectively in the feces compared to the intestine of CR and *ad libitum* fed mice.

The microbiota has been shown to modulate hosts’ amino-acid and GSH metabolism.^43^ Interestingly, the antibiotics treatment of CR mice adjusted (up or down) the levels of GHS-taurine, other taurine conjugates or free taurine, GSH levels, GSTs gene expression and GST enzymatic activity to the levels measured in *ad libitum* fed mice. Importantly, the antibiotics cocktail did not influence the levels in *ad libitum* fed mice. Therefore, we hypothesize that a CR-specific microbiota is responsible for up- and down-regulation of the levels of the compounds and gene expression. This hypothesis was further confirmed by the results of the fecal transplant approach. Moreover, this regulation seems to be intestine-specific and does not affect gene expression and or BAs production in the liver. As expected,^44^ the antibiotics treatment decreased BAs levels in the intestine of *ad libitum* fed mice. Furthermore, we observed a consistent impact of antibiotics on CR-related changes in BAs composition in the liver, plasma, and mucosa of ileum in antibiotics-treated mice. Antibiotics also strongly dampened CR-triggered increase in BAs levels; however, the treatment did not neutralize it completely. Furthermore, transplantation of CR-specific microbiota did not affect BAs levels. Therefore, microbiota contributes to the regulation of CR-triggered increase in the levels of BAs but it is not the pivotal factor.

Interestingly, taurine has been reported to reduce the abundance of phylum *Proteobacteria*, class *Bacilli*, and order *Lactobacillales*, while increasing phylum *Firmicutes*, order *Clostridiales*,^20^ as well as family *Lachnospiraceae*.^21^ In our sequencing results, we found the exact opposite impact of CR on gut microbiota composition. It may seem contradictory considering that the levels of taurine increase in the intestine mucosa during CR. However, due to more efficient uptake during CR^37^ the level of taurine likely decreases in the intestinal lumen, where microbiota resides. Taurine (together with histamine, and spermine) was suggested to shape the host-microbiota interface through activation of *Nlrp6* inflammasome signaling, resulting in intestinal epithelial cell IL-18 secretion and downstream modulation of anti-microbial peptide transcription,^45^ thereby impacting the microbiome composition and risk of auto-inflammation. Still, more research is required to verify if CR-driven changes in taurine levels affect *Nlrp6*.

Besides GST, also other antioxidative proteins were affected in the intestine during CR in this study. Nonetheless, contrary to GST, the expression of antioxidative proteins is reduced, most likely due to a reduction in oxidative stress accompanying CR.^46–49^ Yet, contradictory data concerning oxidative stress in CR have been reported,^50^ e.g. the expression of SOD and other anti-oxidative enzymes in CR remains controversial.^50–52^ Moderate CR has also been reported to increase the activity of Mn-SOD and GSH concentration in the liver.^52^ Therefore, the impact of CR may be tissue-specific.

In summary, we have shown for the first time that microbiota contributes to the intestinal response to CR by modulating GST activity, levels of GSH, taurine, BAs, and CR-stimulated BAs conjugation. Therefore, gut bacteria may be necessary to profit from the beneficial impact of CR. However, the exact meaning behind the CR-driven increase in taurine and its conjugates in the intestine is not clear. Moreover, further investigation is required to unveil the mechanisms of microbial activity during CR.

## Materials and methods

### Animal care and experimental procedures

Male C57Bl/6 mice were purchased from Janvier Labs (Le Genest-Saint-Isle, France) and kept under a 12 h light/12 h dark cycle in standard specific-pathogen-free (SPF) conditions. The mice were given V153x R/M-H auto diet from SSNIFF-Spezialdiäten GmbH (Soest, Germany) and free water access. Mice aged 12 weeks were randomly divided into experimental groups of eight mice, as follows: Ad lib – control *ad libitum* fed; CR – calorie restricted; AT – antibiotics-treated; AT CR – antibiotics-treated and calorie restricted; FT *– ad libitum* fed, antibiotics-treated with fecal microbiota transplant from Ad lib mice; FT CR – *ad libitum* fed, antibiotics-treated with fecal microbiota transplant from CR mice. The groups did not differ significantly in body weight at the beginning of the experimental procedures. The antibiotics treatment and FT were performed and controlled as previously reported.^14^ The mice from the CR and AT-CR groups were submitted to 14 days of CR reduced to ~ 75% of daily food intake. To deplete gut flora, mice from the AT, AT-CR, FT, and FT CR groups were repeatedly gavaged with 200 µl of an antibiotic cocktail (vancomycin .5 g/l, neomycin 1 g/l, ampicillin 1 g/l, metronidazole 1 g/l; all from Sigma-Aldrich, Vienna, Austria). The AT and AT-CR groups were gavaged three times within 14 days of the experimental procedure, and the FT group was gavaged twice: 5 and 3 days before microbiota transplant. After gut flora depletion, the mice from the FT groups were gavaged twice at a 2-day interval with freshly extracted fecal microbiota from CR or *ad libitum* mice. To obtain inoculants, fresh feces were mixed with sterile PBS. The mixture was vortexed and centrifuged for 3 min at 1000 ×g, and the isolated supernatant was immediately gavaged into FT mice. FT mice were killed 7 days after the first gavage.

All animal experimentation protocols were approved by the the Bundesministerium für Wissenschaft, Forschung und Wirtschaft, Referat für Tierversuche und Gentechnik (BMBWF-66.006/0008-V/3b/2018). All experiments were carried out according to animal experimentation Animal Welfare Act guidelines.

### Sequencing the 16S rDNA genes and metataxonomic analysis

The samples for sequencing were processed according to the previously published protocol.^53^ Cecum samples were homogenized in MagNA Pure Bacteria Lysis Buffer from the MagNA Pure LC DNA Isolation Kit III in MagNA Lyser green beads tubes in a MagNA Lyser Instrument (all from Roche, Mannheim, Germany). Next, the samples were mixed with 25 μl lysozyme (100 mg/ml), incubated at 37°C for 30 min, 43.4 μl Proteinase K (20 mg/ml) was added followed by incubation at 65°C overnight. The enzymes were heat-inactivated and 250 μl of supernatant was used for DNA extraction on a MagNA Pure LC 2.0 following the instructions for the MagNA Pure LC DNA Isolation Kit III (all from Roche, Mannheim, Germany). PCRs reactions were run in triplicates using a FastStart High Fidelity PCR system with 5 μl of total DNA, 1x Fast Start High Fidelity Buffer, 1.25 U High Fidelity Enzyme, 200 μM dNTPs, .4 μM primers, and water in 25 μl reaction volume (all reagents from Roche, Mannheim, Germany). The target primers V1-V2: 27 F – AGAGTTTGATCCTGGCTCAG and 375 R – CTGCTGCCTYCCGTA were applied for the amplification of phylogenetic informative hypervariable regions using Illumina adapters for an indexing PCR reaction. The PCR reaction products were checked on an agarose gel and their normalization was performed on a SequalPrep Normalization Plate (LifeTechnologies, Carlsbad, CA, USA). Next, 15 μl of the product was used as a template in a single 50 μl indexing PCR reaction for 8 cycles. Finally, 5 μl of PCR products from each sample were pooled and 30 μl of the library was purified using an agarose gel and the QIAquick gel extraction kit (Qiagen, Hilden, Germany). The obtained library was quantified with QuantiFluor ONE dsDNA Dye on Quantus™ Fluorometer (Promega, Walldorf, Germany), its quality was verified using an Agilent BioAnalyzer 2100 (Waldbronn, Germany) and the 6 pM library was sequenced on a MiSeq desktop sequencer containing 20% PhiX control DNA (Illumina, Eindhoven, Netherlands) with v2 chemistry for 500 cycles.

Raw sequencing data in fastq format was imported in Galaxy web-based platform^54^ and analyzed with the QIIME2 2018.4. The data was preprocessed with DADA2^55^ using default parameters and removing specific primer sequences. The resulting feature representative sequences were classified with the QIIME2 pre-fitted sklearn-based taxonomy classifier against SILVA 16S rRNA database version 132 at 99% identity.^56^

### Protein concentration, activity assays, and total bile acids concentration

The levels of total bile acids, GSH and GSSG, as well as the activity of GR (all from BioVision, Milpitas, CA, USA), were assessed using commercial assay kits according to the manufacturer’s indications.

### Tissue lysate assay

Freshly extracted tissue was lysed in 10x volume (w/v) of lysis buffer (150 mM NaCl, 1% IGEPAL, 50 mM Tris-HCL) by disrupting with syringe and needle. Afterward, 5 µl of the lysate was mixed with 10 µl of 2 M GSH and 2 M taurine and filled up to 200 µl with the lysis buffer. The samples were incubated at 37°C for 20 min.

### Electron spin resonance

ESR assay was performed as previously published.^37^ Shortly, 144 μl of oxygen-free KHB and 6 μl of oxygen-free 10 mM CMH solution were added to 15 μg tissue pieces. The samples were incubated for 60 min in a 37°C shaking incubator, spun down and 100 µl of the solution was used for the measurement. ESR measurements were performed at 150 K in a capillary tube (100 µL) placed into a high sensitivity resonator (Bruker ER 4122SHQE), using an X-band Bruker Elexsys-II E500 EPR spectrometer (Bruker BioSpin GmbH, Rheinstetten, Germany) applying modulation frequency of 100 kHz and a microwave frequency of 9.4 GHz. Spectra were recorded every 20 s, averaging every 10 consecutive spectra. The sweep width was 450 G, the sweep time 20 s, the modulation amplitude 5 G, the center field 3400 G, the microwave power 20 mW, and the resolution was 1024 points. EPR spectra were simulated and the area under the curve was determined by double integration of the spectrum. A reference-free quantitation of the number of spins was performed, as has been described previously.^57^

### Gene expression

Intestine samples were thawed in lysis buffer, disrupted using a syringe and needle. Liver samples were homogenized using Precellys®24 Tissue Homogenizer (Bertin Instruments, Montigny-le-Bretonneux, France). RNA from both types of tissues was isolated using the RNeasy mini kit (Qiagen, Hilden, Germany). For reverse transcription, SuperScript® II Reverse Transcriptase (Invitrogen^TM^, Life Technologies, Carlsbad, CA, USA) was used. Quantitative real-time PCR (qRT-PCR) reactions were performed using the QuantStudio^TM^ 6 Flex Real-Time PCR System with the SYBR Green PCR Master Mix (both from Applied Biosystems, Life Technologies, Carlsbad, CA, USA). The Ct values were normalized to Eef1a1. The results show ΔΔCt averaged for biological replicates for each experimental group. The sequences of the primers used were published before.^37^

### Detection and identification of GSH and taurine conjugates

The detection protocol was performed as published previously.^37^ Briefly, 7–10 mg of intestinal mucosa samples were homogenized using syringe and needle and disrupted in five thawing and freezing cycles. Next, nine times the volume of ethanol absolute −20°C was added, vortexed for 30 s and samples were centrifuged for 10 min at 18,000 g at 4°C and the supernatants were analyzed by LCMS in negative modus using an LCMS-8040 Liquid Chromatograph Mass Spectrometer (Shimadzu Corporation, Kyoto, Japan) with an Atlantis T3 3 μm column (2.1x150 mm, Waters, Milford, MA, USA). The column temperature was 40°C. The mobile phases consisted of .1% formic acid in water (eluent A) and .1% formic acid in acetonitrile (eluent B). The gradient was maintained at an initial 5% B for 2.5 min, to 20% B at 8 min, and was set back to 5% B at 9 min with a hold for one minute.

Identification of conjugates was performed as previously.^37^ Shortly, standards of GSH and taurine (both from Sigma-Aldrich, St. Louis, MO, USA) were prepared in 70% ethanol. Standards were fragmented in negative modus using an LCMS-8040 Liquid Chromatograph Mass Spectrometer (Shimadzu Corporation, Kyoto, Japan) with an Atlantis T3 3 μm column (2.1x150 mm, Waters, Milford, MA, USA). The mobile phases and the gradient was conforming with the detection protocol. The fragmentation pattern was compared to METLIN´s database. Precursor ions in the tested samples were compared to fragmentation patterns of GSH and taurine. The exact mass of the selected precursor ions was measured using an Ultimate 3000 (Thermo Fischer Scientific, Waltham, MA, US) and a micrOTOF-Q II (Bruker Daltonics, Bremen, Germany) with an Atlantis T3 3 μm column (2.1x150 mm, Waters, Milford, MA, USA) kept at 40°C. The mobile phases and the gradient were set the same as for GSH, taurine, and conjugated detection. The mass and fragmentation pattern of chosen precursors was verified using the METLIN database. The compounds identified as GSH or taurine conjugates were previously published.^37^

### Bile acid analysis

As reported previously,^37^ bile acids were measured with LCMS. Liver samples were weighted in Precellys homogenizing tubes with 1.4 mm ceramic beads, and nine times the volume of methanol absolute at −20°C was added. Samples were homogenized in the Precellys 24 Tissue Homogenizer (Bertin Instruments, Montigny-le-Bretonneux, France) twice for 15 s at 5000 rpm, vortexed for 30 s, and centrifuged for 10 min at 5000 g at 4°C. The supernatants were transferred to new 1.5 ml Eppendorf tubes, and, to remove the remaining debris, the centrifugation step was repeated this time at 12,000 g. Supernatants were transferred into new Eppendorf tubes and after second centrifugation, supernatants were directly transferred into HPLC vials. Intestinal samples were processed as described above in the method for GSH and taurine analysis. Plasma samples (50 μl) were extracted with 150 μl MeOH, vortexed for 30 s, and shaken continuously for 10 min with a laboratory rocker. 100 μl of the supernatant was evaporated, re-dissolved in 50 μl methanol, and transferred into an HPLC vial. After the extraction, samples were handled alike. Samples (10 μl) were analyzed by LCMS in positive modus using an LCMS-8040 Liquid Chromatograph Mass Spectrometer (Shimadzu Corporation, Kyoto, Japan) with an Atlantis T3 3 μm column (2.1 × 150 mm, Waters, Milford, MA, USA). The column temperature was 30°C. The mobile phase A consisted of water and eluent B was acetonitrile/methanol (3/1, v/v), both containing .1% formic acid and a concentration of 20 mM ammonium acetate. The gradient was maintained from an initial 30% B for 5 min, to 100% B at 25 min, which was kept constant for 20 min. Afterward, the composition was set back to the initial ratio of 30% B within 2 min, followed by 10 min of re-equilibration.

## Supplementary Material

Supplemental Material

## Data Availability

The microbiota datasets generated during and analyzed during the current study are available in the European Nucleotide Archive [https://www.ebi.ac.uk/ena/browser/view/PRJEB46415] repository.

## Data availability statement

The authors confirm that the data supporting the findings of this study are available within the article and its supplementary materials.

## Statistics

For analysis of statistical differences between four groups, one-way ANOVA with Bonferroni correction for multiple testing was applied using SPSS Statistics 26 (IBM Corp., Armonk, NY, USA). Data sets with two groups of seven to nine biological replicates were compared using a two-sided Student’s t-test and a *p*-value lower than .05 was considered statistically significant.

## References

[1] Martin R, Makino H, Cetinyurek Yavuz A, Ben-Amor K, Roelofs M, Ishikawa E, Kubota H, Swinkels S, Sakai T, Oishi K, et al. Early-life events, including mode of delivery and type of feeding, siblings and gender, shape the developing gut microbiota. PloS One. 2016;11(6):e0158498. doi:10.1371/journal.pone.0158498. 27362264 PMC4928817

[2] Yatsunenko T, Rey FE, Manary MJ, Trehan I, Dominguez-Bello MG, Contreras M, Magris M, Hidalgo G, Baldassano RN, Anokhin AP, et al. Human gut microbiome viewed across age and geography. Nature. 2012;486(7402):222–14. doi:10.1038/nature11053. 22699611 PMC3376388

[3] Ianiro G, Tilg H, Gasbarrini A. Antibiotics as deep modulators of gut microbiota: between good and evil. Gut. 2016;65(11):1906–1915. doi:10.1136/gutjnl-2016-312297. 27531828

[4] Gupta VK, Paul S, Dutta C. Geography, ethnicity or subsistence-specific variations in human microbiome composition and diversity. Front Microbiol. 2017;8:1162. doi:10.3389/fmicb.2017.01162. 28690602 PMC5481955

[5] Rothschild D, Weissbrod O, Barkan E, Kurilshikov A, Korem T, Zeevi D, Costea PI, Godneva A, Kalka IN, Bar N, et al. Environment dominates over host genetics in shaping human gut microbiota. Nature. 2018;555(7695):210–215. doi:10.1038/nature25973. 29489753

[6] van Hylckama Vlieg JE, Veiga P, Zhang C, Derrien M, Zhao L. Impact of microbial transformation of food on health - from fermented foods to fermentation in the gastro-intestinal tract. Curr Opin Biotechnol. 2011;22(2):211–219. doi:10.1016/j.copbio.2010.12.004. 21247750

[7] Zhang C, Zhang M, Wang S, Han R, Cao Y, Hua W, Mao Y, Zhang X, Pang X, Wei C, et al. Interactions between gut microbiota, host genetics and diet relevant to development of metabolic syndromes in mice. ISME J. 2010;4(2):232–241. doi:10.1038/ismej.2009.112. 19865183

[8] Alvarez Y, Glotfelty LG, Blank N, Dohnalova L, Thaiss CA. The microbiome as a circadian coordinator of metabolism. Endocrinology. 2020;161(6). doi:10.1210/endocr/bqaa059. PMC7899566 32291454

[9] Santacruz A, Marcos A, Wärnberg J, Martí A, Martin-Matillas M, Campoy C, Moreno LA, Veiga O, Redondo-Figuero C, Garagorri JM, et al. Interplay between weight loss and gut microbiota composition in overweight adolescents. Obesity (Silver Spring). 2009;17(10):1906–1915. doi:10.1038/oby.2009.112. 19390523

[10] Zhang C, Li S, Yang L, Huang P, Li W, Wang S, Zhao G, Zhang M, Pang X, Yan Z, et al. Structural modulation of gut microbiota in life-long calorie-restricted mice. Nat Commun. 2013;4(1):2163. doi:10.1038/ncomms3163. 23860099 PMC3717500

[11] Zheng X, Wang S, Jia W. Calorie restriction and its impact on gut microbial composition and global metabolism. Front Med. 2018;12(6):634–644. doi:10.1007/s11684-018-0670-8. 30446879

[12] Ruiz A,Cerdó T, Jáuregui R, Pieper DH, Marcos A, Clemente A, García F, Margolles A, Ferrer M, Campoy C, Suárez A. One-year calorie restriction impacts gut microbial composition but not its metabolic performance in obese adolescents. Environ Microbiol. 2017;19:1536–1551. doi:10.1111/1462-2920.13713. 28251782

[13] Tanca A, Abbondio M, Palomba A, Fraumene C, Marongiu F, Serra M, Pagnozzi D, Laconi E, Uzzau S. Caloric restriction promotes functional changes involving short-chain fatty acid biosynthesis in the rat gut microbiota. Sci Rep. 2018;8(1):14778. doi:10.1038/s41598-018-33100-y. 30283130 PMC6170429

[14] Duszka K, Ellero-Simatos S, Ow GS, Defernez M, Paramalingam E, Tett A, Ying S, König J, Narbad A, Kuznetsov VA, et al. Complementary intestinal mucosa and microbiota responses to caloric restriction. Sci Rep. 2018;8(1):11338. doi:10.1038/s41598-018-29815-7. 30054525 PMC6063912

[15] Wang S, Huang M, You X, Zhao J, Chen L, Wang L, Luo Y, Chen Y. Gut microbiota mediates the anti-obesity effect of calorie restriction in mice. Sci Rep. 2018;8(1):13037. doi:10.1038/s41598-018-31353-1. 30158649 PMC6115465

[16] Duszka K, Wahli W. Enteric Microbiota–gut–brain axis from the perspective of nuclear receptors. Int J Mol Sci. 2018;19(8):2210. doi:10.3390/ijms19082210. PMC6121494 30060580

[17] Foley MH, O’Flaherty S, Barrangou R, Theriot CM, Knoll LJ. Bile salt hydrolases: gatekeepers of bile acid metabolism and host-microbiome crosstalk in the gastrointestinal tract. PLoS Pathog. 2019;15(3):e1007581. doi:10.1371/journal.ppat.1007581. 30845232 PMC6405046

[18] Ridlon JM, Kang DJ, Hylemon PB. Bile salt biotransformations by human intestinal bacteria. J Lipid Res. 2006;47(2):241–259. doi:10.1194/jlr.R500013-JLR200. 16299351

[19] Devkota S, Wang Y, Musch MW, Leone V, Fehlner-Peach H, Nadimpalli A, Antonopoulos DA, Jabri B, Chang EB. Dietary-fat-induced taurocholic acid promotes pathobiont expansion and colitis in Il10−/− mice. Nature. 2012;487(7405):104–108. doi:10.1038/nature11225. 22722865 PMC3393783

[20] Yu H, Guo Z, Shen S, Shan W. Effects of taurine on gut microbiota and metabolism in mice. Amino Acids. 2016;48(7):1601–1617. doi:10.1007/s00726-016-2219-y. 27026373

[21] Fang H,Meng F, Piao F, Jin B, Li M, Li W. Effect of taurine on intestinal microbiota and immune cells in peyer’s patches of immunosuppressive mice. Adv Exp Med Biol. 2019;1155:13–24. doi:10.1007/978-981-13-8023-5_2. 31468382

[22] Shimizu M, Zhao Z, Ishimoto Y, Satsu H. Dietary taurine attenuates dextran sulfate sodium (DSS)-induced experimental colitis in mice. Adv Exp Med Biol. 2009;643:265–271. doi:10.1007/978-0-387-75681-3_27. 19239157

[23] Zhao Z, Satsu H, Fujisawa M, Hori M, Ishimoto Y, Totsuka M, Nambu A, Kakuta S, Ozaki H, Shimizu M, et al. Attenuation by dietary taurine of dextran sulfate sodium-induced colitis in mice and of THP-1-induced damage to intestinal Caco-2 cell monolayers. Amino Acids. 2008;35(1):217–224. doi:10.1007/s00726-007-0562-8. 17619120

[24] Gondo Y, Satsu H, Ishimoto Y, Iwamoto T, Shimizu M. Effect of taurine on mRNA expression of thioredoxin interacting protein in Caco-2 cells. Biochem Biophys Res Commun. 2012;426(3):433–437. doi:10.1016/j.bbrc.2012.08.116. 22960072

[25] Kim C, Cha YN. Taurine chloramine produced from taurine under inflammation provides anti-inflammatory and cytoprotective effects. Amino Acids. 2014;46(1):89–100. doi:10.1007/s00726-013-1545-6. 23933994

[26] Marcinkiewicz J, Mak M, Bobek M, Biedroń R, Białecka A, Koprowski M, Kontny E, Maśliński W. Is there a role of taurine bromamine in inflammation? Interactive effects with nitrite and hydrogen peroxide. Inflamm Res. 2005;54(1):42–49. doi:10.1007/s00011-004-1322-9. 15723204

[27] Niu X, Zheng S, Liu H, Li S. Protective effects of taurine against inflammation, apoptosis, and oxidative stress in brain injury. Mol Med Rep. 2018;18:4516–4522. doi:10.3892/mmr.2018.9465. 30221665 PMC6172387

[28] Chupel MU, Minuzzi LG, Furtado G, Santos ML, Hogervorst E, Filaire E, Teixeira AM. Exercise and taurine in inflammation, cognition, and peripheral markers of blood-brain barrier integrity in older women. Appl Physiol Nutr Metab. 2018;43(7):733–741. doi:10.1139/apnm-2017-0775. 29474803

[29] Silva LA, Silveira PCL, Ronsani MM, Souza PS, Scheffer D, Vieira LC, Benetti M, De Souza CT, Pinho RA. Taurine supplementation decreases oxidative stress in skeletal muscle after eccentric exercise. Cell Biochem Funct. 2011;29(1):43–49. doi:10.1002/cbf.1716. 21264889

[30] Thirupathi A, Freitas S, Sorato HR, Pedroso GS, Effting PS, Damiani AP, Andrade VM, Nesi RT, Gupta RC, Muller AP, et al. Modulatory effects of taurine on metabolic and oxidative stress parameters in a mice model of muscle overuse. Nutrition. 2018;54:158–164. doi:10.1016/j.nut.2018.03.058. 29982143

[31] Bucolo C,Fidilio A, Platania CBM, Geraci F, Lazzara F, Drago F. Antioxidant and osmoprotecting activity of taurine in dry eye models. J Ocul Pharmacol Ther. 2018;34(1–2):188–194. doi:10.1089/jop.2017.0008. 28771380

[32] Lee DS, Jo HG, Kim MJ, Lee H, Cheong SH. Antioxidant and anti-stress effects of taurine against electric foot-shock-induced acute stress in rats. Adv Exp Med Biol. 2019;1155:185–196. doi:10.1007/978-981-13-8023-5_17. 31468397

[33] Trachtman H, Barbour R, Sturman JA, Finberg L. Taurine and osmoregulation: taurine is a cerebral osmoprotective molecule in chronic hypernatremic dehydration. Pediatr Res. 1988;23(1):35–39. doi:10.1203/00006450-198801000-00008. 3340441

[34] Kong B, Wang L, Chiang JYL, Zhang Y, Klaassen CD, Guo GL. Mechanism of tissue-specific farnesoid X receptor in suppressing the expression of genes in bile-acid synthesis in mice. Hepatology. 2012;56(3):1034–1043. doi:10.1002/hep.25740. 22467244 PMC3390456

[35] Inagaki T, Choi M, Moschetta A, Peng L, Cummins CL, McDonald JG, Luo G, Jones SA, Goodwin B, Richardson JA, et al. Fibroblast growth factor 15 functions as an enterohepatic signal to regulate bile acid homeostasis. Cell Metab. 2005;2(4):217–225. doi:10.1016/j.cmet.2005.09.001. 16213224

[36] Renga B, Mencarelli A, Cipriani S, D’Amore C, Carino A, Bruno A, Francisci D, Zampella A, Distrutti E, Fiorucci S, et al. The bile acid sensor FXR is required for immune-regulatory activities of TLR-9 in intestinal inflammation. PLoS One. 2013;8(1):e54472. doi:10.1371/journal.pone.0054472. 23372731 PMC3555871

[37] Gregor A, Pignitter M, Fahrngruber C, Bayer S, Somoza V, König J, Duszka K. Caloric restriction increases levels of taurine in the intestine and stimulates taurine uptake by conjugation to glutathione. J Nutr Biochem. 2021;96:108781. doi:10.1016/j.jnutbio.2021.108781. 34022385

[38] Ebert MN, Beyer-Sehlmeyer G, Liegibel UM, Kautenburger T, Becker TW, Pool-Zobel BL. Butyrate induces glutathione S-transferase in human colon cells and protects from genetic damage by 4-hydroxy-2-nonenal. Nutr Cancer. 2001;41(1–2):156–164. doi:10.1080/01635581.2001.9680627. 12094619

[39] Fabbiano S, Suárez-Zamorano N, Chevalier C, Lazarević V, Kieser S, Rigo D, Leo S, Veyrat-Durebex C, Gaïa N, Maresca M, et al. Functional gut microbiota remodeling contributes to the caloric restriction-induced metabolic improvements. Cell Metab. 2018;28(6):907–921 e907. doi:10.1016/j.cmet.2018.08.005. 30174308 PMC6288182

[40] Sayin SI, Wahlström A, Felin J, Jäntti S, Marschall H-U, Bamberg K, Angelin B, Hyötyläinen T, Orešič M, Bäckhed F, et al. Gut microbiota regulates bile acid metabolism by reducing the levels of tauro-beta-muricholic acid, a naturally occurring FXR antagonist. Cell Metab. 2013;17(2):225–235. doi:10.1016/j.cmet.2013.01.003. 23395169

[41] Anderson CM, Howard A, Walters JR, Ganapathy V, Thwaites DT. Taurine uptake across the human intestinal brush-border membrane is via two transporters: h+-coupled PAT1 (SLC36A1) and Na+- and Cl−-dependent TauT (SLC6A6). J Physiol. 2009;587(4):731–744. doi:10.1113/jphysiol.2008.164228. 19074966 PMC2669967

[42] Boyer JL. Bile formation and secretion. Compr Physiol. 2013;3:1035–1078. doi:10.1002/cphy.c120027. 23897680 PMC4091928

[43] Mardinoglu A, Shoaie S, Bergentall M, Ghaffari P, Zhang C, Larsson E, Bäckhed F, Nielsen J. The gut microbiota modulates host amino acid and glutathione metabolism in mice. Mol Syst Biol. 2015;11(10):834. doi:10.15252/msb.20156487. 26475342 PMC4631205

[44] Theriot CM, Bowman AA, Young VB, Ellermeier CD. Antibiotic-induced alterations of the gut microbiota alter secondary bile acid production and allow for clostridium difficile spore germination and outgrowth in the large intestine. mSphere. 2016;1(1). doi:10.1128/mSphere.00045-15. PMC4863611 27239562

[45] Levy M, Thaiss C, Zeevi D, Dohnalová L, Zilberman-Schapira G, Mahdi J, David E, Savidor A, Korem T, Herzig Y, et al. Microbiota-modulated metabolites shape the intestinal microenvironment by regulating NLRP6 inflammasome signaling. Cell. 2015;163(6):1428–1443. doi:10.1016/j.cell.2015.10.048. 26638072 PMC5665753

[46] Barja G. Aging in vertebrates, and the effect of caloric restriction: a mitochondrial free radical production-DNA damage mechanism? Biol Rev Camb Philos Soc. 2004;79(2):235–251. doi:10.1017/S1464793103006213. 15191224

[47] Forster MJ, Sohal BH, Sohal RS. Reversible effects of long-term caloric restriction on protein oxidative damage. J Gerontol A Biol Sci Med Sci. 2000;55(11):B522–529. doi:10.1093/gerona/55.11.B522. 11078084

[48] Lambert AJ, Merry BJ. Lack of effect of caloric restriction on bioenergetics and reactive oxygen species production in intact rat hepatocytes. J Gerontol A Biol Sci Med Sci. 2005;60(2):175–180. doi:10.1093/gerona/60.2.175. 15814858

[49] Lambert AJ, Portero-Otin M, Pamplona R, Merry BJ. Effect of ageing and caloric restriction on specific markers of protein oxidative damage and membrane peroxidizability in rat liver mitochondria. Mech Ageing Dev. 2004;125(8):529–538. doi:10.1016/j.mad.2004.06.002. 15336910

[50] Agarwal S, Sharma S, Agrawal V, Roy N. Caloric restriction augments ROS defense in S. cerevisiae, by a Sir2p independent mechanism. Free Radic Res. 2005;39(1):55–62. doi:10.1080/10715760400022343. 15875812

[51] Mesquita A, Weinberger M, Silva A, Sampaio-Marques B, Almeida B, Leao C, Costa V, Rodrigues F, Burhans WC, Ludovico P, et al. Caloric restriction or catalase inactivation extends yeast chronological lifespan by inducing H2O2 and superoxide dismutase activity. Proc Natl Acad Sci U S A. 2010;107(34):15123–15128. doi:10.1073/pnas.1004432107. 20696905 PMC2930563

[52] Stankovic M, Mladenovic D, Ninkovic M, Vucevic D, Radosavljevic TT. Effects of caloric restriction on oxidative stress parameters. Gen Physiol Biophys. 2013;32(2):277–283. doi:10.4149/gpb_2013027. 23682026

[53] Klymiuk I, Bilgilier C, Stadlmann A, Thannesberger J, Kastner MT, Högenauer C, Püspök A, Biowski-Frotz S, Schrutka-Kölbl C, Thallinger GG, et al. The human gastric microbiome is predicated upon infection with helicobacter pylori. Front Microbiol. 2017;8:2508. doi:10.3389/fmicb.2017.02508. 29312210 PMC5735373

[54] Afgan E, Baker D, Batut B, van den Beek M, Bouvier D, Čech M, Chilton J, Clements D, Coraor N, Grüning BA, et al. The Galaxy platform for accessible, reproducible and collaborative biomedical analyses: 2018 update. Nucleic Acids Res. 2018;46(W1):W537–W544. doi:10.1093/nar/gky379. 29790989 PMC6030816

[55] Callahan BJ,McMurdie PJ, Rosen MJ, Han AW, Johnson AJ, Holmes SP. DADA2: high-resolution sample inference from illumina amplicon data. Nat Methods. 2016;13(7):581–583. doi:10.1038/nmeth.3869. 27214047 PMC4927377

[56] Yilmaz P,Parfrey LW, Yarza P, Gerken J, Pruesse E, Quast C, Schweer T, Peplies J, Ludwig W, Glöckner FO. The SILVA and “All-species Living Tree Project (LTP)” taxonomic frameworks. Nucleic Acids Res. 2014;42(D1):D643–648. doi:10.1093/nar/gkt1209. 24293649 PMC3965112

[57] Zaunschirm M, Pignitter M, Kienesberger J, Hernler N, Riegger C, Eggersdorfer M, Somoza V. Contribution of the ratio of tocopherol homologs to the oxidative stability of commercial vegetable oils. Molecules. 2018;23(1):206. doi:10.3390/molecules23010206. PMC6017329 29351234

