# Supplementary material for: Microbial contribution to the caloric restriction-triggered regulation of the intestinal levels of glutathione transferases, taurine, and bile acid
Source: Gut Microbes. 2021 Oct 25;13(1):1992236. doi: 10.1080/19490976.2021.1992236 (PMC8547879; doi:10.1080/19490976.2021.1992236)
Supplement: Supplemental Material [file KGMI_A_1992236_SM2304.zip › supplementary figures.pdf]

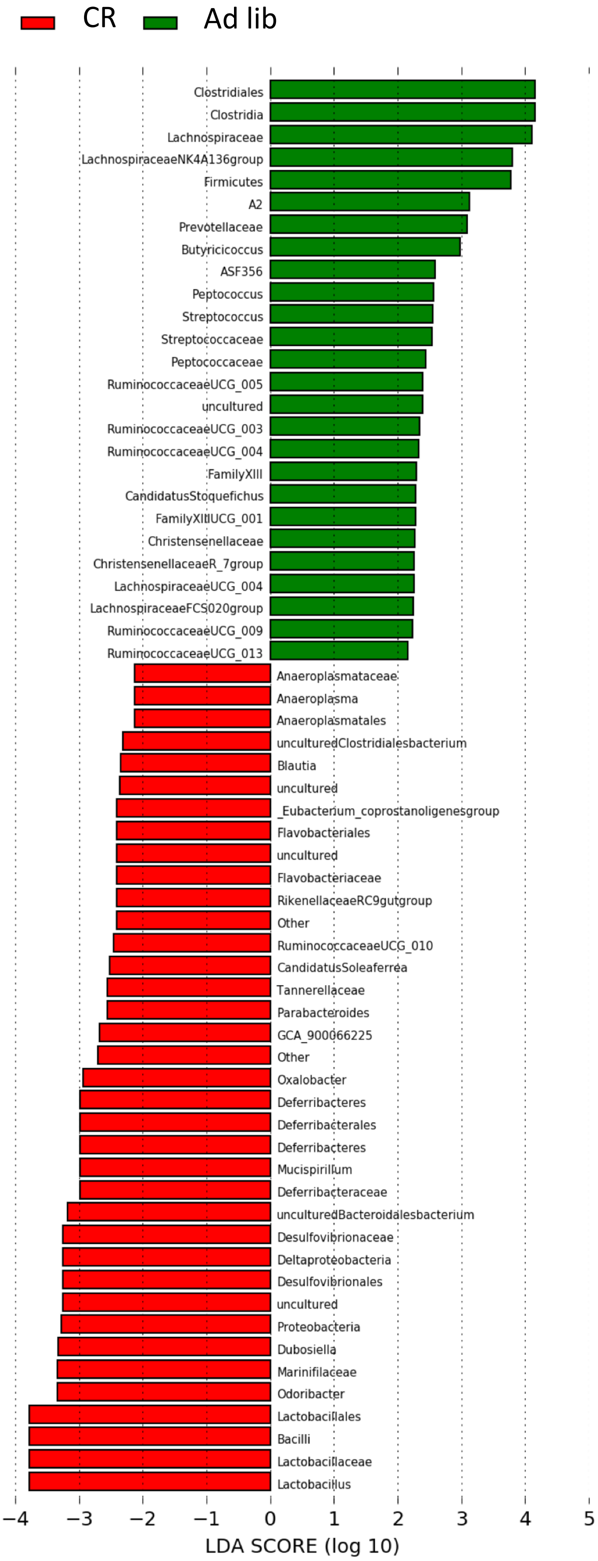

Supplementary figure 1: Barchart for the significantly changed taxa in CR and *ad libitum* mice cecum ranking them accordingly to their effect size and associating them with the class with the highest median. The figure presents LEfSe results with LDA score bigger than 2 and p-value smaller than 0.05.

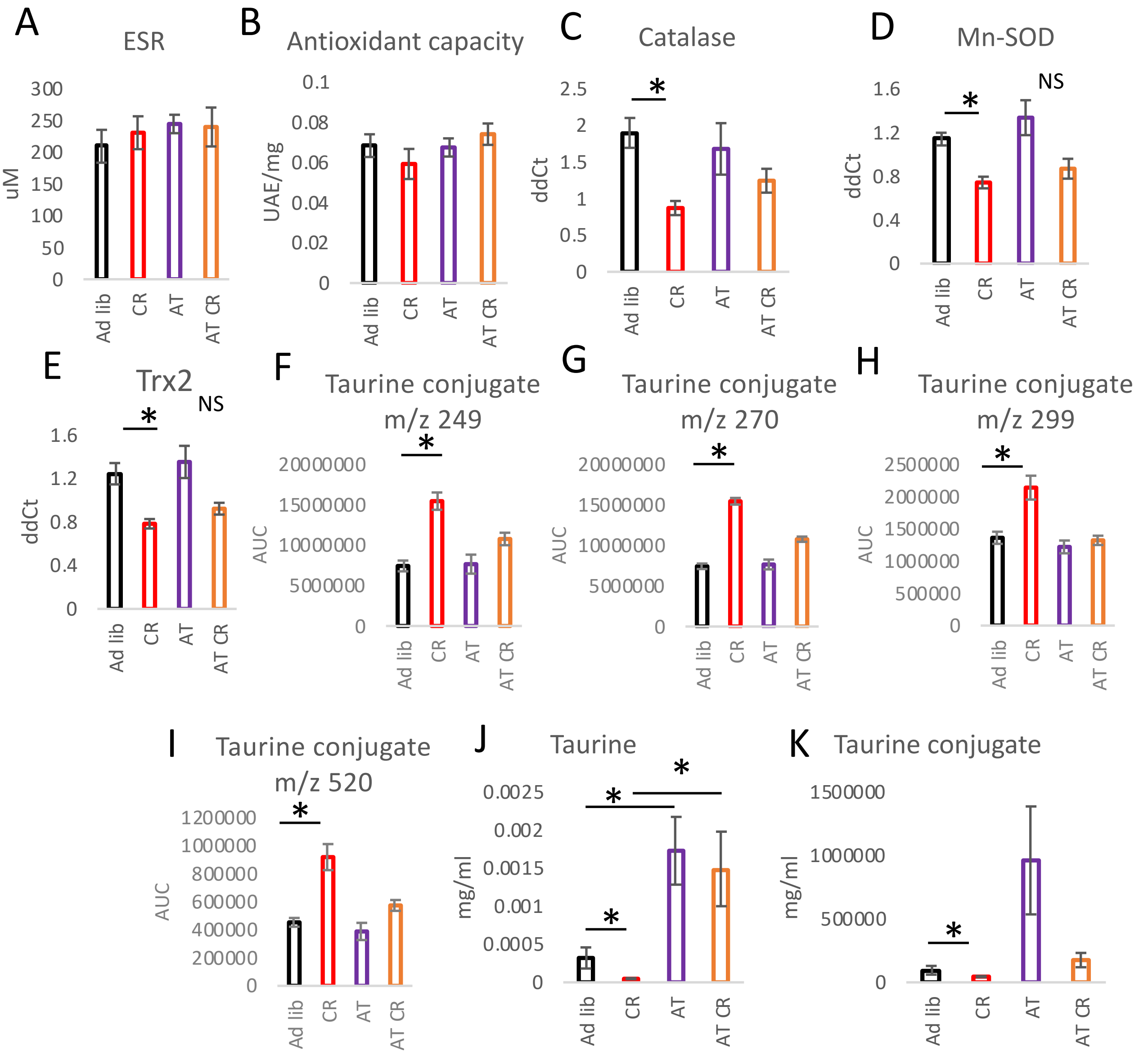

Supplementary figure 2: Electron spin resonance (ESR) was applied to assess the levels of reactive oxygen species in the mucosa of the jejunum (A). Antioxidant capacity was measured in the jejunum mucosa applying commercial assay (B). The mRNA expression of catalase, manganese superoxide dismutase (Mn-Sod), and thioredoxin 2 (Trx2) was measured using qRT-PCR (C-E). The levels of taurine conjugates were measured in the ileum mucosa (F-I). The levels of taurine and its conjugates were measured in feces (J-K). Statistical significance between the experimental groups was evaluated using ANOVA with Bonferroni correction for multiple testing; n=5-8;* $p<0.05$. Data are presented as mean±SEM.

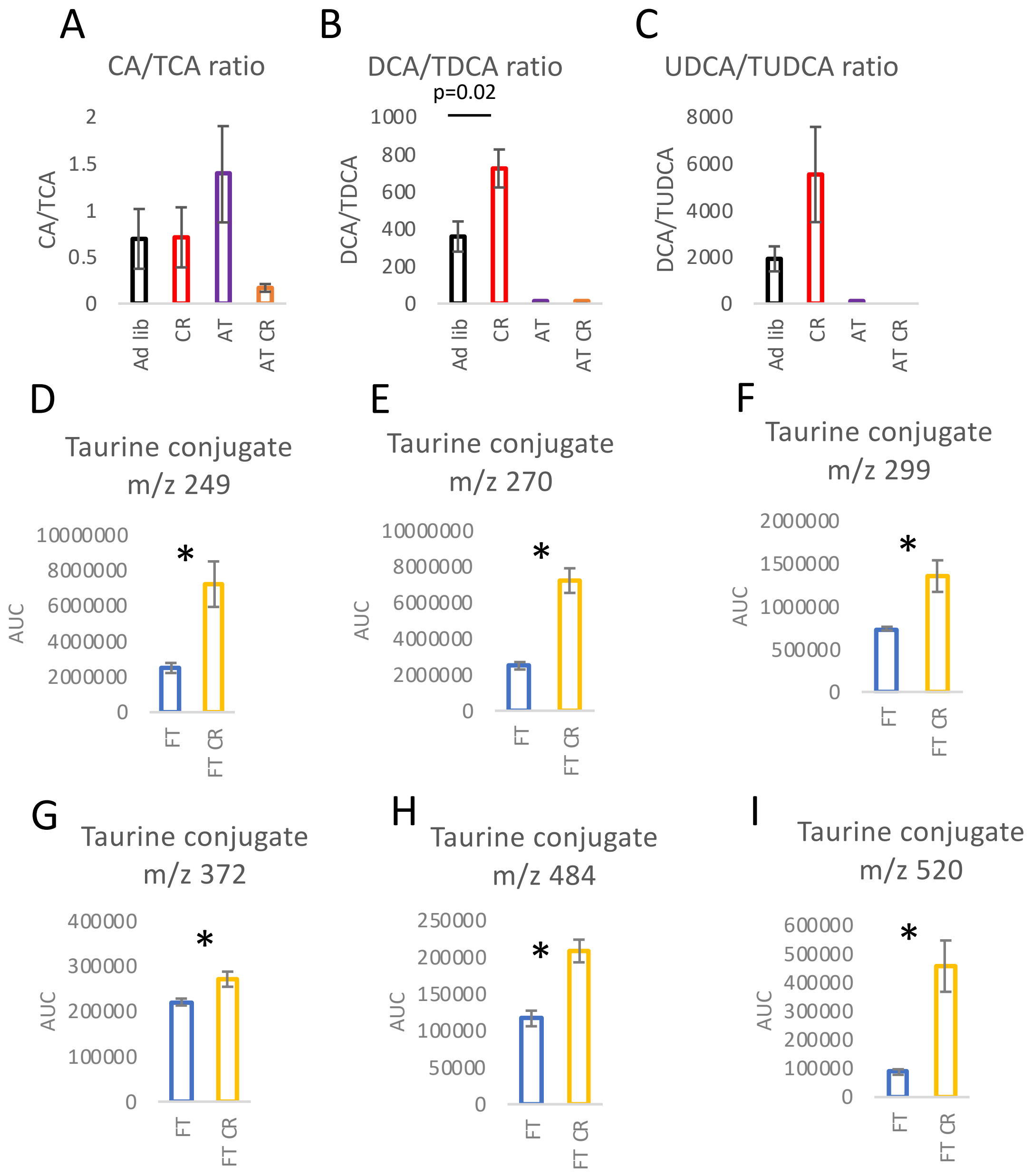

Supplementary figure 3: The ratio of CA to TCA (A), DCA to TDCA (B), and UDCA to TUDCA (C) was calculated based on BAS concentration measured in the feces. The levels of taurine conjugates were measured in the ileum mucosa (D-I). Statistical significance between CR and *ad libitum* groups was evaluated using ANOVA (panels A-C) or two-tailed Student's t-tests (panels D-I); n=6-8; *p<0.05. Data are presented as mean±SEM.
