# Supplementary material for: Microbial contribution to the caloric restriction-triggered regulation of the intestinal levels of glutathione transferases, taurine, and bile acid
Source: Gut Microbes. 2021 Oct 25;13(1):1992236. doi: 10.1080/19490976.2021.1992236 (PMC8547879; doi:10.1080/19490976.2021.1992236)
Supplement: Supplemental Material [file KGMI_A_1992236_SM2304.zip › supplementary table1.pdf]

Supplementary table 1. Levels of bile acids (BA) in the ileum, liver and plasma of all experimental groups. Mass to charge ratio (m/z) of BAs and the corresponding main fragment ions is indicted under the BA's name.

a indicates p<0,05 compared to *ad libitum* group; b symbols p<0,05 compared to CR group; c corresponds p<0,05 compared to AT group.

	**CA**<br>426.34>373.2	**TCA**<br>533.33>516.2	**TLCA**<br>501.35>466.2	**TDCA**<br>517.35>500.2	**TUDCA**<br>517.32>464.2	**DHCA**<br>420.34>385.2	**UDCA**<br>410.18>357.2	**DCA**<br>410.35>357.3
**Ileum** (ng/mg)								
**Ad lib**	412.158<br>± 112.826	4956.162<br>± 912.660	0.262<br>± 0.065 **c**	20.964<br>± 4.408 **c**	12.928<br>± 2.796	0.009<br>± 0.007	45.972<br>± 8.965 **c**	20.941<br>± 5.310
**CR**	1377.039<br>± 407.470 **c**	7802.993<br>± 2044.440	1.352<br>± 0.374 **a, c**	63.341<br>± 18.925 **c**	23.547<br>± 6.483	0.191<br>± 0.080 **a, c**	394.353<br>± 194.971	172.848<br>± 84.690
**AT**	290.260<br>± 170.817 **b**	4063.148<br>± 970.010	0.043<br>± 0.030 **a, b**	3.055<br>± 1.361 **a, b**	21.620<br>± 6.884	0 **b**	9.180<br>± 6.155 **a**	3.472<br>± 2.444 **a**
**AT CR**	266.930<br>± 52.856 **b**	10819.282<br>± 1821.59 **a**	0.005<br>± 0.004 **a, b**	0.625<br>± 0.553 **a, b**	16.246<br>± 4.140	0 **b**	0 **a**	0 **a**
**FT**	946.474<br>± 283.762	6154.043<br>± 1884.080 **c**	0.829<br>± 0.235 **c**	48.767<br>± 23.182	25.089<br>± 10.895	0.148<br>± 0.072	98.998<br>± 40.983	66.664<br>± 22.485 **c**
**FT CR**	656.874<br>± 265.965 **b**	5790.882<br>± 114.274	0.466<br>± 0.148 **b, c**	25.663<br>± 7.555 **b, c**	16.965<br>± 4.536	0.059<br>± 0.055	89.157<br>± 41.815	42.200<br>± 17.101
**Liver** (ng/mg)								
**Ad lib**	1.254<br>± 0.214	1115.958<br>± 187.309	0.170<br>± 0.059	17.259<br>± 2.520 **c**	7.074<br>± 0.779 **c**	0	0	0
**CR**	13.459<br>± 4.374 **a, c**	3145.440<br>± 77.963 **a**	0.968<br>±0.210 **a, c**	82.894<br>±11.808 **a, c**	24.452<br>± 4.479 **a**	0.0002<br>± 0.0001	2.201<br>± 2.206	0
**AT**	2.790<br>± 0.925 **b**	2111.583<br>± 427.343	0.046<br>± 0.022 **b**	5.528<br>± 1.729 **a, b**	26.253<br>± 5.837 **a**	0	0	0
**AT CR**	8.105<br>± 4.172	7577.759<br>± 2535.667 **a**	0 **a, b**	10.324<br>± 4.912 **b**	57.445<br>± 19.285 **a**	0	0	0
**FT**	3.989<br>± 2.211	1279.555<br>± 297.031 **b**	0.393<br>± 0.110 **b, c**	33.935<br>± 7.877 **b**	11.377<br>± 3.266 **b, c**	0	0	0.944<br>± 0.842
**FT CR**	2.297<br>± 0.716 **b**	1566.077<br>± 377.371	0.215<br>± 0.045 **b, c**	24.420<br>± 4.637 **b**	9.313<br>± 1.954 **b, c**	0	0	2.829<br>± 2.667
**Plasma** (μg/ml)								
**Ad lib**	0.860<br>± 0.411	260.653<br>± 155.81	0.014<br>± 0.009	6.231<br>± 3.923	1.488<br>± 0.872	0	0	0
**CR**	43.814<br>± 24.450	3333.975<br>± 1866.322	0.508<br>± 0.333	171.482<br>± 104.738	23.546<br>± 14.992	0.1758<br>± 0.127	4.513<br>± 1.935 **a**	1.464<br>± 0.808
**AT**	0.997<br>± 0.583	1277.599<br>± 835.319	0.004<br>± 0.003	1.718<br>± 0.950	13.749<br>± 9.715	0	0.254<br>± 0.237	0
**AT CR**	13.662<br>± 5.377 **a, b, c**	8190.064<br>± 2677.830 **a, c**	0.014<br>± 0.006	36.290<br>± 12.636 **c**	76.051<br>± 26.801 **a**	0.033<br>± 0.031	0.124<br>± 0.116	0
**FT**	0.856<br>± 0.405	285.849<br>± 263.236	0.02<br>± 0.019	9.344<br>± 8.768	1.929<br>± 1.756	0	0.567<br>± 0.359	0.177<br>± 0.166
**FT CR**	2.572<br>± 1.441	745.258<br>± 686.973	0.047<br>± 0.041	16.683<br>± 14.633	4.703<br>± 3.681	0.026<br>± 0.024	0.693<br>± 0.539	0.268<br>± 0.253

**Feces** (μg/ml)								
**Ad lib**	0.107 ± 0.052	0.166 ± 0.036	0.0002 ± 0 **c**	0.0037 ± 0.000 **c**	0.002 ± 0	0.0007 ± 0.0001	1.978 ± 0.301 **c**	0.974 ± 0.151 **c**
**CR**	0.043 ± 0.013 **c**	0.079 ± 0.012 **a**	0.0002 ± 0 **c**	0.00332 ± 0.000 **c**	0.001 ± 0	0.0008 ± 0.0002	4.174 ± 0.549 **a, c**	2.034 ± 0.260 **a, c**
**AT**	0.458 ± 0.146 **b**	0.492 ± 0.246	0.0001 ± 0 **a, b**	0.0009 ± 0.0002 **a, b**	0.002 ± 0	0.0006 ± 0.0013	0.007 ± 0.003 **a, b**	0.002 ± 0.001 **a, b**
**AT CR**	0.113 ± 0.033 **c**	0.822 ± 0.182 **a, b**	0.0001 ± 0 **a, b**	0.0005 ± 0.0001 **a, b**	0.004 ± 0.001 **b**	0.007 ± 0.0003	0 **a, b, c**	0.0003 ± 0.0003 **a, b**
**FT**	0.034 ± 0.0164 **c**	0.675 ± 0.221 **b**	0.0001 ± 0 **a, b**	0.0008 ± 0.0001 **a, b**	0.002 ± 0	0.0003 ± 0.005 **c**	0.013 ± 0.009 **a, b**	0.007 ± 0.005 **a, b**
**FT CR**	0.007 ± 0.004 **b, c**	0.7476 ± 0.103 **a, b**	0.0001 ± 0	0.0009 ± 0.0001 **a, b**	0.004 ± 0 **a, b, c**	0.0002 ± 0 **a, b**	0.001 ± 0.0004 **a, b, c**	0 **a, b**
